# Coronary calcium scoring on contrast‐enhanced spectral coronary computed tomography angiography using a calcium‐specific algorithm

**DOI:** 10.1002/mp.70238

**Published:** 2025-12-25

**Authors:** L. R. Koetzier, P. M. Tetteroo, E. Encinas Vargas, A. M. R. Schilham, M. J. W. Greuter, E. Langzam, B. K. Velthuis, D. Suchá, N. R. van der Werf

**Affiliations:** ^1^ Department of Radiology and Nuclear Medicine University Medical Center Utrecht Utrecht The Netherlands; ^2^ Department of Radiology University Medical Center Groningen University of Groningen Groningen The Netherlands; ^3^ Clinical Science, Philips Healthcare, Best Utrecht The Netherlands

## Abstract

**Background:**

Coronary artery calcium (CAC) scoring requires a non‐contrast scan in addition to contrast‐enhanced coronary CT angiography (CCTA), increasing radiation dose and scan time. Virtual non contrast (VNC) methods from spectral CT may obviate the CAC CT scan, but available methods underestimate CAC scores and show low detectability. Although VNC‐based CAC scoring is feasible via conversion factors, it misses low‐density calcifications, requiring improved VNC methods.

**Purpose:**

This study evaluates a self‐developed CAC‐preserving VNC (TrueCa) method to improve detectability and quantification of CAC on CCTA.

**Methods:**

An artificial hollow coronary artery containing five cylindrical calcifications of varying density calcium hydroxyapatite (75–800 mg/cc) was filled with 0%, 50%, 100% (400 Hounsfield units) and 150% of a clinical iodine concentration. The phantom was scanned on dual‐layer spectral CT with a CAC scoring and CCTA protocol with five repetitions. CAC CT reconstruction followed Agatston protocol. CCTA scans were reconstructed at thick (3 mm) and thin (0.67 mm) slices using (1) commercially available VNC and (2) self‐developed TrueCa algorithm, generated via linear separation of spectral data. Non‐overlapping 95% confidence intervals between Agatston scores (AS) from the CAC protocol and CCTA protocol indicated significant differences. Volume scores (VS) were compared to physical calcification volume.

**Results:**

For AS, thick‐slice VNC CCTA underestimated AS by 30%–65% across all CAC densities, failing to detect CAC below 400 mg/cc. TrueCa CCTA detected all calcifications without significant difference with CAC CT for all CAC densities. Thin‐slice compared to thick‐slice TrueCa significantly increased AS in low‐density CAC.

For VS, TrueCa CCTA showed less underestimation than VNC CCTA across all densities except for highest‐density calcifications. VS accuracy improved by up to 40% using TrueCa over VNC in medium to lowest‐density calcifications.

**Conclusion:**

In this phantom study, TrueCa CCTA demonstrated superior performance in calcium detection and quantification across most CAC densities, showing potential as a reliable alternative to CAC CT.

## 1 INTRODUCTION

Detection and risk assessment of coronary artery disease (CAD) typically involves a two‐step computed tomography (CT) approach; a true non‐contrast (TNC) coronary artery calcium (CAC) scoring CT followed by contrast‐enhanced coronary lumen stenosis evaluation using coronary CT angiography (CCTA), as per clinical guidelines.^1^, ^2^ CAC scoring provides a reproducible and widely accessible tool for assessing the risk of short and long term major adverse cardiovascular events (MACE).^3^ The Agatston method, introduced in 1990 and updated in 2007, still remains the clinical reference standard for CAC quantification, relying on non‐contrast CT at specific parameters; 120 kilovolt peak (kVp) acquisition, filtered back projection (FBP) reconstruction, and 3 or 2.5 mm slice thickness images.^4^, ^5^, ^6^ Conducting this separate CAC scan, however, accounts for approximately 20% of the total radiation exposure and extends the overall coronary exam by an additional 5 min.^7^, ^8^ In an effort to reduce radiation exposure and patient burden, and move from a two‐step to a one‐step CT approach, previous studies have attempted to derive CAC scores directly from CCTA scans.^8^, ^9^ These studies often report a strong correlation between CAC scores obtained from CAC CT and CCTA, although the level of agreement is limited due to underestimated scores. Calcium and iodine share overlapping CT attenuation values with conventional CT, making it impractical to separate CAC from the contrast lumen.

Multi‐energy CT systems, however, enable the capture of separate photon energy spectra (i.e. spectral information), allowing for material decomposition and improved tissue characterization. Dual‐layer spectral CT (DLCT) systems provide spectral information without any compromise in, amongst others, temporal resolution, radiation dose, spatial resolution, and field‐of‐view. This in turn gives opportunities for improved and more reliable CAC assessment. Using spectral information, virtual non‐contrast (VNC) images can be calculated and reconstructed from CCTA, offering the potential to eliminate the need for separate CAC scans. In addition, the typically lower noise levels in CCTA scans allow for the reconstruction of thinner low‐noise slices, which may enhance the detection of small calcifications without increasing false positives.^10^ While promising, published studies investigating VNC as a substitute for CAC generally have reported an underestimation of CAC scores, necessitating a conversion factor that limits generalizability.^11^, ^12^, ^13^ VNC reconstructions use water and iodine as basis materials to model voxel content. As calcium is not represented in this basis, CAC may be partly modelled as iodine, leading to its suppression on VNC images. This study proposes a novel, self‐developed CAC‐preserving VNC (TrueCa) method, with the aim to more accurately quantify the CAC score from CCTA.

## 2 METHODS

### 2.1 Phantom set‐up

**FIGURE 1:**
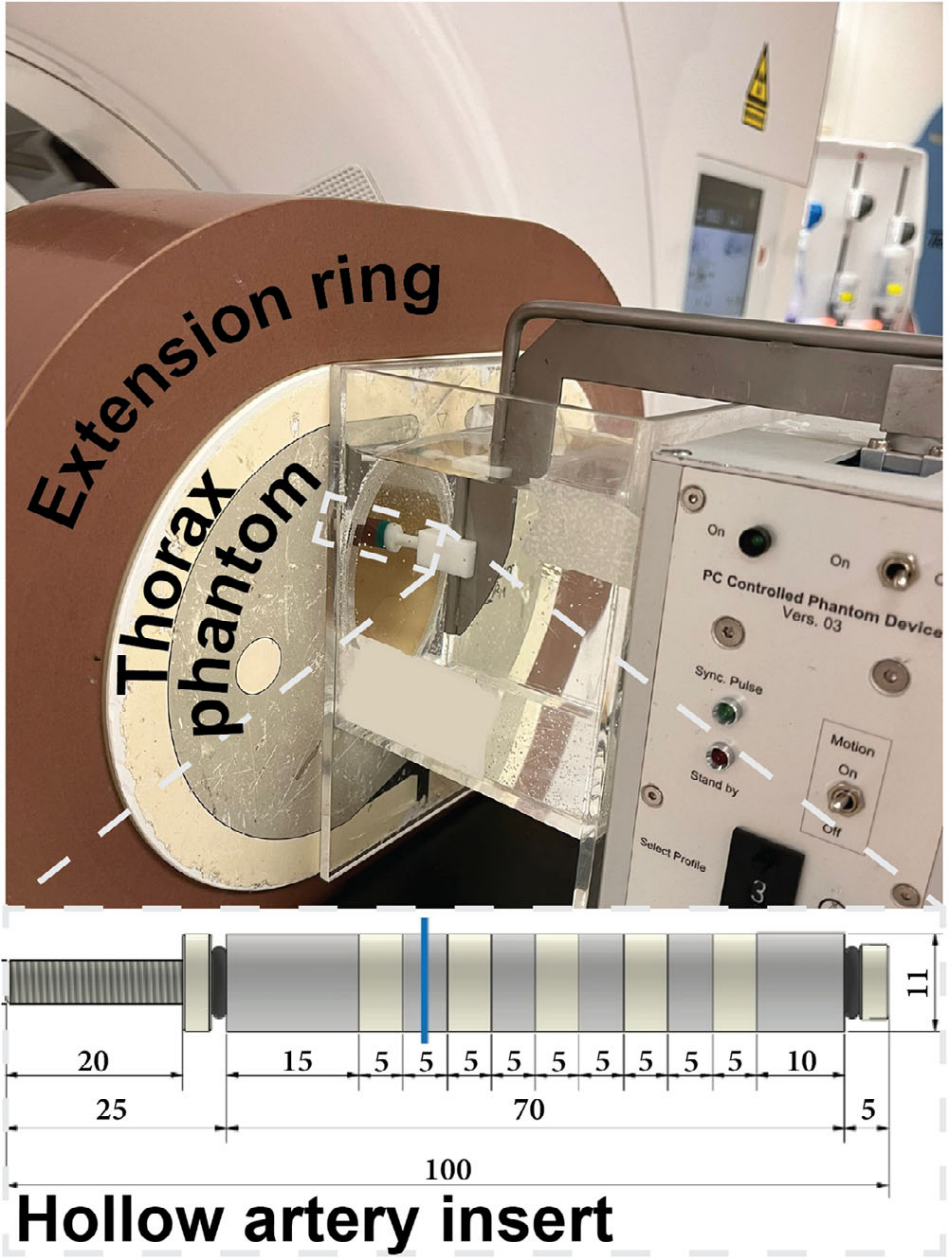
Representation of the phantom scanning setup. A thorax phantom, equipped with an extension ring, was positioned within the CT gantry. A hollow artery insert, containing solid water and five calcium hydroxyapatite calcifications, was placed within a water bath inside the thorax phantom. The artery was mounted on a motion controller capable of simulating coronary movement, although this functionality was not utilized in the present study. All dimensions of the hollow artery are provided in millimeters.

To simulate a cardiac patient, an anthropomorphic thoracic CT phantom (QRM‐thorax, PTW Freiburg) was used. A fat‐equivalent extension ring (Extension Ring Fat L, PTW Freiburg) was attached to increase the phantom from 300 mm × 200 mm to 400 mm × 300 mm in order to simulate a large patient size (body‐mass index (BMI) ≥ 25 kg/m^2^).^6^ A static cardiac insert containing CAC was used to evaluate CAC quantification. For this, a hollow artificial artery (Calcium Artery, PTW Freiburg) was placed in a water‐filled compartment in the thoracic phantom. The artificial artery contained five concentric calcifications with same dimensions (inner diameter 5 mm, outer diameter 11 mm, length 5 mm, physical volume 377 mm^3^), but different calcium hydroxyapatite (CaHA) densities (75, 100, 200, 400, and 800 mg CaHA/cc; theoretically 96, 129, 259, 521, and 1044 Hounsfield units (HU) at 70 keV, and 0, 0, 302, 603, and 603 Agatston score points, respectively) (Figure 1). Calcium rings maximize partial‐volume and blooming artifacts, representing a worst‐case scenario for calcium scoring. For TNC CT acquisitions, the artery was filled with a mixture of water and glucose with the same attenuation as blood (40 HU) and contained a 0% contrast medium dose. For CCTA acquisitions, diluted iodinated contrast agent (Iopromide, 300 mg I/mL, Bayer Inc.) was added to the mixture to simulate 50%, 100%, and 150% clinical contrast medium doses, resulting in approximately 185, 390, and 590 HU at 120 kVp. The artery was oriented parallel to the *z*‐axis of the CT system.

### 2.2 Data acquisition and reconstruction

The phantom was sequentially scanned on a DLCT system (Spectral CT7500, Philips Healthcare). Two clinical routine acquisition protocols were used: one for TNC CAC CT and another for CCTA and TNC coronary CT (CCT) (Table 1). The CAC CT was acquired at a lower tube current and reconstructed with a different reconstruction algorithm than CCTA/CCT. TNC CCT refers to the non‐contrast (0% contrast medium) scan following the CCTA protocol, enabling comparison with contrast‐enhanced CCTA under identical acquisition and reconstruction settings. The entire scanning process was repeated five times for each contrast medium concentration, obtaining five CAC CT (reference) acquisitions, 15 CCTA acquisitions, and five CCT acquisitions. After each repetition, the coronary artery insert was manually repositioned (approximately 2 degrees, 2 mm) to simulate inter‐scan variability. CAC CT, CCTA, and TNC CCT images were reconstructed at slice thickness/increment 3.0/1.5 mm using FBP and iterative reconstruction with kernel CB, as per Agatston method and CCTA protocol. Additionally, CCTA and TNC CCT images were reconstructed at 0.67/0.34 mm slices to potentially allow for detectability of lower‐density calcifications.

**TABLE 1:** Coronary artery calcium (CAC) CT and (true non contrast (TNC)) coronary CT angiography (CCTA) acquisition and reconstruction parameters.

Parameter	CAC CT	CCTA/TNC CCT
Technique	Axial	Axial
Tube voltage [kVp]	120	120
Reference tube current product [mAs]^*^	65	202
CTDI_vol_ [mGy]	5.0	15.6
Collimation [mm]	128 × 0.625	128 × 0.625
Field of view [mm]	220	220
Rotation time [s]	0.27	0.27
Pitch	‐	‐
Slice thickness/increment [mm]	3.0/1.5	0.67/0.4 & 3.0/1.5
Reconstruction filter	CB	CB
Matrix size [pixels]	512	512
Reconstruction	iDose4 level 0^**^	iDose4 level 4
Acquisitions	5	20

* Based on automatic exposure control index (Dose Right Index; DRI, Philips Healthcare) of 13 and 22 for CAC CT and CCTA.

** iDose4 level 0 is equivalent to reconstruction with filtered back projection.

**FIGURE 2:**
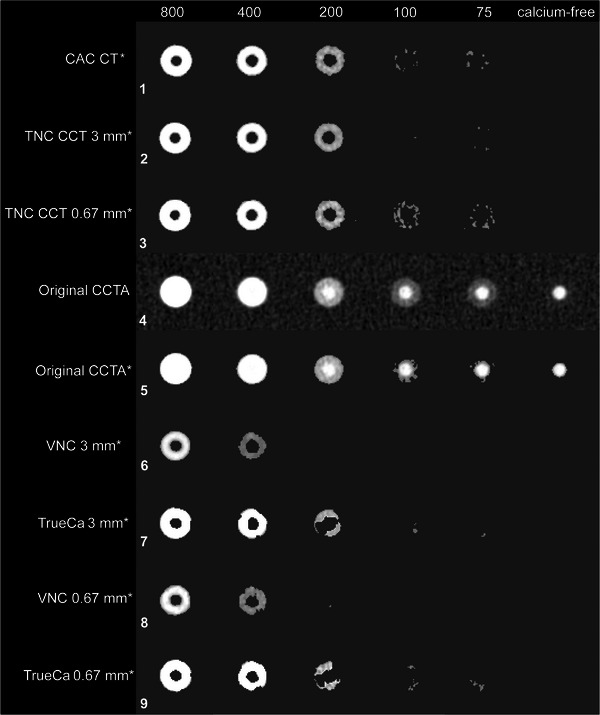
Example images comparing coronary artery calcium (CAC) scoring CT, true non contrast coronary CT (TNC CCT), coronary CT angiography (CCTA) and two virtual non‐contrast (VNC) methods. The top three rows show in vitro CAC CT and TNC CCT (3 mm and 0.67 mm) of calcifications with varying coronary artery calcium (CAC) densities in mg/cc. The fourth to ninth row show images from contrast‐enhanced CCTA. In the sixth and eighth row, the CT values of calcifications were suppressed by the calcium non‐specific VNC method, resulting in undetected calcifications with medium and low density (200, 100, and 75 mg CaHA/cc). In contrast, the seventh and ninth row applies the calcium‐preserving TrueCa approach, which preserves CT values of CAC, improving detection of calcifications of all densities. An asterisk (*) is used to indicate that calcium thresholding is applied at 130 Hounsfield units (HU) (WL, 70 HU; WW, 600 HU).

Two algorithms were used to generate VNC images from the same physical CCTA and CCT scans. In the first method, traditional VNC reconstructions were obtained directly from commercially available software (IntelliSpace Portal 15.0, Philips Healthcare). This VNC algorithm removes the iodine contribution from spectral base materials, and then produces a virtual monoenergetic image at 70 keV from the adjusted base materials (Figure 2, sixth and eighth row).^14^

The developed method as proposed in this report, TrueCa, separates iodine from calcium by utilizing spectral base images corresponding to the photoelectric effect and Compton scatter. These specific spectral base images form the foundation for spectral reconstructions in Philips CT systems and are not commercially available, but were provided by Philips Research. The TrueCa method analyzes the HU values from these two base images on a voxel‐by‐voxel basis. Each voxel is classified using a linear decision boundary, formulated as:

(1) $$\begin{array}{cc} \mathrm{HU}\left(\mathrm{Photoelectric}\;\mathrm{effect}\right) & \leq \mathrm{HU}\left(\mathrm{Compton}\;\mathrm{scatter}\right)\\ & \;\times \mathrm{slope}+\mathrm{intercept} \end{array}$$

**FIGURE 3:**
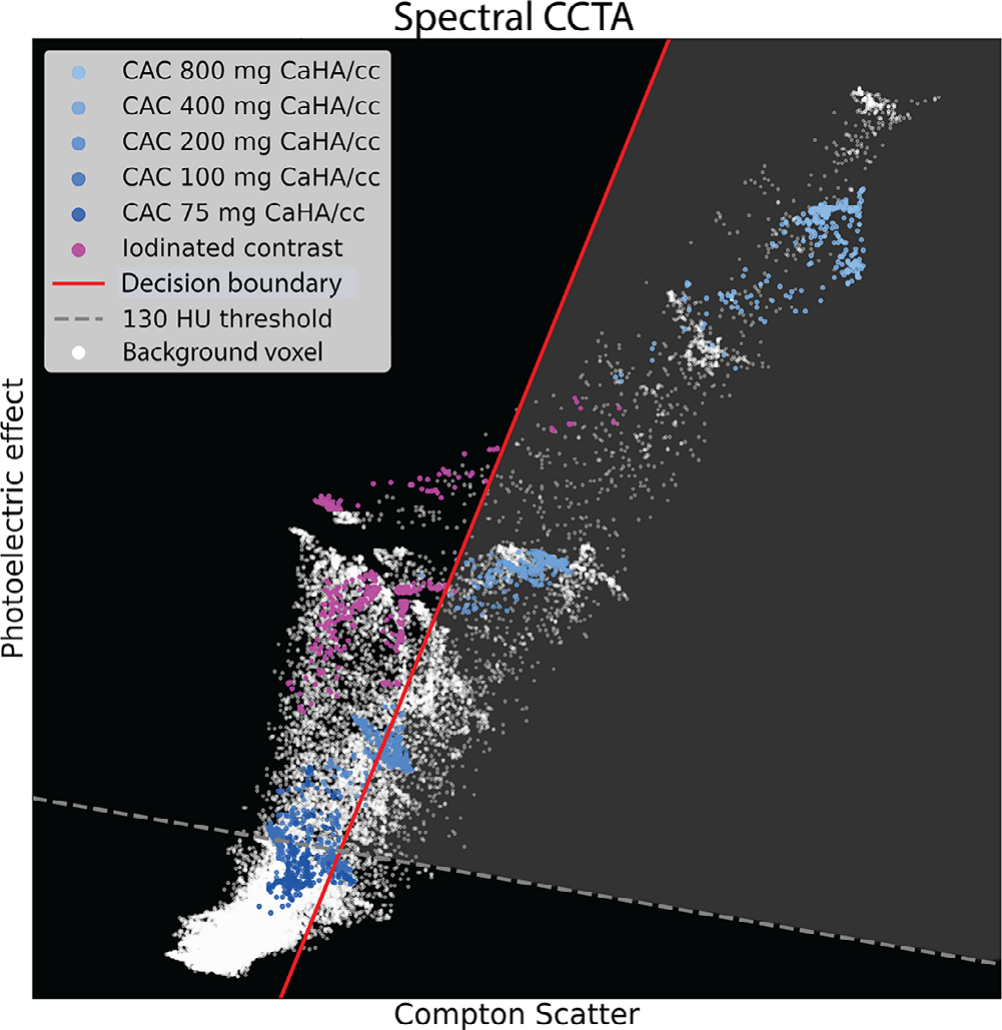
Illustration of spectral scatter plot from in vitro coronary CT angiography (CCTA) of an 100% clinical contrast dose iodine‐filled hollow artery containing five calcifications with varying calcium hydroxyapatite densities. Using regions of interest in the image domain while avoiding edge‐effects, iodinated contrast (pink) and calcifications (blue) of different densities are highlighted in the spectral scatter plot. Background voxels are displayed in white and may belong to the phantom edges. To separate calcium from iodine, data points positioned below the decision boundary (red line) are included in the calcium mask. Additionally, a threshold of 130 Hounsfield units is applied for coronary artery calcium scoring. The light‐grey area corresponds to the voxels used for calcium masking for TrueCa.

The slope and intercept of the linear decision boundary were determined through iterative optimization using differential evolution, specifically by minimizing the sum of absolute errors per calcification between Agatston scores derived from TrueCa CCTA scans and those from TNC CAC CT scans used in this study. The differential evolution process ran for up to 1000 iterations and terminated when the relative improvement of the best solution fell below 0.1%, resulting in a slope of 3.69 HU(Photoelectric effect)/HU(Compton scatter) and an intercept of −3361 HU(Photoelectric effect). For visualization purposes, voxel distributions and the separation line can be depicted in a two‐dimensional spectral scatter plot (Figure 3). When inequality (1) is satisfied, the voxel (**r)** is identified as predominantly calcium‐containing and is added to a binary calcium mask (*M*):

(2) $$M\;\left(r\right)=\left\{\begin{array}{cc} 1, & \mathrm{if}\;\mathrm{inequality}\;\left(1\right)\;\mathrm{is}\;\mathrm{satisfied}\\ 0, & \mathrm{otherwise} \end{array}\right.\;$$

The mask is applied to the conventional 120 kVp CCTA image (*I*_Conv_) to generate the TrueCa CCTA images (Figure 2, seventh and nineth rows), where voxels within the calcium mask retain their attenuation and all others are set to 0 HU:

(3) $$I_{\mathrm{TrueCa}}\left(r\right)=M\left(r\right)\cdot I_{\mathrm{Conv}}\left(r\right)$$

### 2.3 CAC quantification

CAC score, by means of Agatston score and volume scores, were automatically measured for each calcification in the hollow artery using a validated Python script.^15^ Calcium was quantified on 120 kVp images from the reference TNC CAC CT, TNC CCT, VNC CCTA, and TrueCa CCTA reconstructions, based on a threshold of at least 0.5 mm^2^ in‐plane area and 130 HU.^6^ A 3 mm equivalent Agatston score was calculated to account for the non‐standard thickness and increment. For volume score comparison, physical calcification volume (377 mm^3^) served as the reference for each calcification.

### 2.4 Statistical analysis

For CAC quantification analysis by Agatston score, in each calcification, contrast concentration, and slice thickness category the mean Agatston score and 95% confidence intervals (CI) for all reconstructions were calculated based on the five repetitions. Agatston score comparisons between VNC CCTA, TrueCa CCTA, and the respective reference Agatston score of CAC CT were made for each density category, contrast concentration and slice thickness, with non‐overlapping 95% confidence intervals indicating significant differences. Volume scores are presented as relative differences from the physical calcification volume. Analyses were performed using the SciPy statistics package version 1.15.1 in Python.

### 2.5 Generative AI and large language models

Artificial intelligence tools were used solely to improve the clarity, grammar, and readability of the manuscript text. No AI tools were used for study design, data collection, data analysis, image generation, or interpretation of results.

## 3 RESULTS

### 3.1 CAC Agatston quantification

Reference Agatston scores for each calcification obtained from the CAC CT and TNC CCT are presented in Table 2. Compared to the reference CAC CT, thick‐slice TNC CCT showed statistically comparable Agatston scores for medium‐ to high‐density calcifications (≥200 mg CaHA/cc). In contrast, for low‐density calcifications (75 and 100 mg CaHA/cc), the Agatston scores were on average 5 and 8 points lower than those from CAC CT, respectively. Agatston scores derived from thin‐slice TNC CCT were significantly overestimated by 27% to 295%, except in very high‐density calcifications.

**TABLE 2:** Calculated true non contrast (TNC) Agatston scores using a coronary artery calcium (CAC) CT protocol and a coronary CT (CCT) protocol.

CAC density	CAC CT	TNC CCT‐0.67 mm	TNC CCT‐3.0 mm
800 mg CaHA/cc	795 (738 ‐ 851)	821 (788 ‐ 853)	780 (730 ‐ 830)
400 mg CaHA/cc	525 (473 ‐ 577)	670 (654 ‐ 686)	515 (459 ‐ 572)
200 mg CaHA/cc	262 (230 ‐ 295)	371 (365 ‐ 379)	220 (211 ‐ 229)
100 mg CaHA/cc	20 (17 ‐ 23)	79 (75 ‐ 84)	12 (10 ‐ 15)
75 mg CaHA/cc	6 (5 ‐ 7)	23 (20 ‐ 26)	1 (0 ‐ 1)
No calcium	0 (0 ‐ 0)	0 (0 ‐ 0)	0 (0 ‐ 0)

*Note*: The 95% confidence intervals of Agatston scores are given between parentheses.

Abbreviation: CaHA = calcium hydroxyapatite.

**FIGURE 4:**
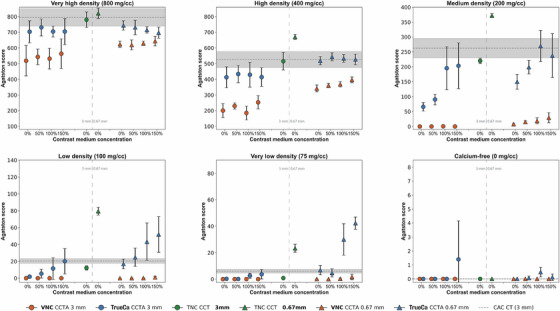
The Agatston scores derived from VNC CCTA, TrueCa CCTA, and TNC CCT were compared using two slice thicknesses, with the 95% confidence intervals of CAC CT serving as the reference standard (grey bars). The results were grouped based on calcification density and contrast medium concentration, with results expressed as mean values alongside their 95% confidence intervals. VNC, virtual non contrast; TNC, true non contrast; CCT(A), coronary CT (angiography); CAC, coronary artery calcium.

Agatston scores per calcification density of VNC CCTA and TrueCa CCTA reconstructions are displayed in Figure 4. Thick‐slice VNC CCTA (orange circles) produced significantly lower Agatston scores than CAC CT, with averaged differences of 256 and 308 points for the 800 and 400 mg CaHA/cc calcifications, respectively, and no detectable calcifications below 400 mg CaHA/cc. In contrast, thick‐slice TrueCa CCTA (blue circles) detected calcifications in all densities and demonstrated statistically comparable Agatston scores with CAC CT across all densities and contrast concentrations (100% and 150%). At lower densities (< 200 mg CaHA/cc), the Agatston scores of 0% (TNC) and 50% contrast concentrations were significantly lower than the reference. Low nonzero Agatston scores were observed in the calcium‐free region at 150% contrast concentration, but this was not significantly different from the reference.

With thin‐slice VNC CCTA (orange triangles), the Agatston scores were significantly lower than those obtained from CAC CT, differing by 166, 157, and 191 points on average for 800, 400, and 200 mg CaHA/cc calcifications, respectively. Calcifications below 200 mg CaHA/cc were not detected, and no differences were found in the calcium‐free region. Conversely, all calcifications were detected using thin‐slice TrueCa CCTA (blue triangles), and Agatston scores were statistically comparable to the CAC CT across all calcifications, but not across all contrast concentrations. The Agatston score at higher contrast concentrations were closer to the Agatston score of thin‐slice TNC CCT (green triangle). In general, Agatston scores of thin‐slice TrueCa CCTA were higher than thick‐slice TrueCa CCTA, which was especially notable in the lower density calcifications.

### 3.2 CAC volume quantification

**FIGURE 5:**
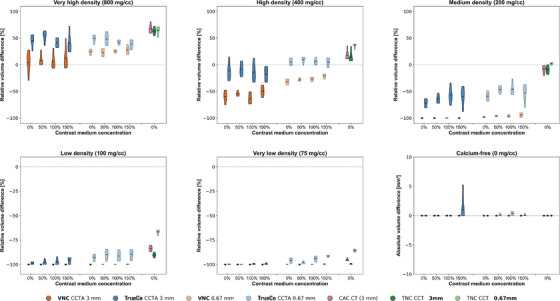
The volume scores derived from VNC CCTA, TrueCa CCTA, TNC CCT, and CAC CT were compared with physical volume of the calcification (377 mm^3^) using two slice thicknesses. The relative volume difference for calcifications is displayed in violin plots, which show the probability density of volume scores estimated with a Gaussian kernel density estimator and normalization for each VNC method, slice thickness, and contrast concentration. In regions without calcium the absolute volume difference is displayed. Results were grouped based on calcification density and contrast medium concentration. VNC, virtual non contrast; TNC, true non contrast; CCT(A), coronary CT (angiography); CAC, coronary artery calcium.

The relative volume score difference per calcification varied across acquisition, reconstruction, and calcification (Figure 5). For very high‐density calcifications, both VNC CCTA and TrueCa CCTA overestimated the physical reference volume of calcifications by up to 28% and 38%, respectively, while CAC CT overestimated the reference by 67%. The highest agreement in volume score for TrueCa CCTA was observed in the high‐density calcification (400 mg CaHA/cc). In medium‐ to very low‐density calcifications, TrueCa CCTA improved the volume score agreement by up to 40% compared to VNC CCTA. In these calcifications, thin‐slice reconstructions also resulted in higher volume scores compared to thick‐slice reconstructions. Overall, these findings indicate a progressive increase in volume underestimation with decreasing calcification density, particularly in the VNC method.

## 4 DISCUSSION

In this phantom study, the presented TrueCa algorithm demonstrated high calcium detectability and strong agreement in Agatston score compared to the reference CAC CT, across a range of calcification densities as well as in calcium‐free regions. Only in low‐density calcifications (≤100 mg/cc), TrueCa‐derived Agatston scores tended to be overestimated when thin‐slice reconstructions and higher concentrations of contrast medium were used. In contrast, the commercially available VNC algorithm left medium‐ to very low‐density calcifications undetected and consistently underestimated Agatston score significantly across all density categories.

For CAC volume quantification, TrueCa CCTA overestimated the reference calcification volume of very high‐density calcifications, although overestimation was less than with CAC CT. In contrast, volume scores were underestimated for medium‐ to very low‐density calcifications compared to the reference volume. Across all density levels, VNC CCTA consistently yielded lower volume scores than TrueCa CCTA and CAC CT, reflecting its limited applicability for CAC scoring.

Several previous studies evaluating CAC non‐specific VNC methods have reported similar underestimation of CAC compared to CAC CT. For instance, Nadjiri et al. demonstrated a high correlation between Agatston score obtained from VNC CCTA and CAC CT, but with limited agreement. To address this discrepancy, the authors proposed a proportionality factor of 2.3 to correct for Agatston score underestimation.^16^ However, the present study demonstrates that medium‐ to very low‐density calcifications often remain undetected in VNC CCTA, a limitation that cannot be resolved by a simple proportionality factor. While deep learning–based approaches have shown potential for CAC quantification on CCTA,^17^, ^18^, ^19^ their dependence on spatial context makes them susceptible to variability in patient anatomy and imaging protocols. In contrast, spectral data provide a more direct characterization of material composition. This enables more reliable differentiation between substances such as calcium and iodine, which can share similar polychromatic but distinct monochromatic attenuation properties. By eliminating dependence on spatial context, our method may offer enhanced robustness and generalizability across diverse anatomical variations and acquisition parameters. Additionally, our classification approach preserves the HU values of calcified voxels, enabling more accurate CAC scoring.

Accurate quantification of low‐density calcifications in thick‐slice TrueCa CCTA remains challenging with the Agatston method. However, the use of thinner slice reconstructions increased Agatston and volume scores across all imaging types (VNC CCTA, TrueCa CCTA, and TNC CCT), indicating that slice thickness plays a critical role in improving calcium detectability, particularly in small and low‐density lesions.^20^, ^21^ While adjusted thresholds in thick‐slice TrueCa CCTA may further enhance sensitivity, such modifications must be carefully balanced against specificity.

Thinner slice reconstructions, made possible by the higher radiation dose of CCTA, offer a marked improvement in out‐of‐plane spatial resolution by reducing partial volume effects, thereby enhancing the detection of subtle calcifications that may remain undetected when using conventional 3 mm slices. This increased sensitivity unmasks sub‐threshold calcifications, which, although their prognostic clinical significance remains to be fully established, may alter patient risk stratification by revealing early atherosclerotic changes not apparent on thicker slices.^10^

Although the calcifications in our phantom were relatively large and already reached the 130 HU threshold using 3 mm slices, this study still observed higher Agatston scores using thin slices. The source of this result is likely twofold: increased detection of calcium in the boundary slices of the calcification and greater HU variability due to noise in thin slices more often elevates the Agatston weighting factor of the calcification.^6^

Nonetheless, the enhanced sensitivity achieved with thinner slices may lead to increased Agatston scores relative to standard protocols, potentially resulting in higher clinical risk classification and management recommendations. Additionally, the conventional threshold of 130 HU, originally defined based on thick slice reconstructions, may require re‐evaluation in the context of thin‐slice imaging.^10^ Therefore, while the application of thinner slices holds promise for improving the accuracy of CAC scoring, its implications for reproducibility and clinical decision‐making warrant further systematic investigation. One important implication of using smaller slice thickness is that the current thick‐slice Agatston score has been widely applied across major large cohort studies, leading to extensive prognostic data for this method. In contrast, there is limited data available for newer scoring methods that may offer improved sensitivity, robustness, and dose efficiency.^10^, ^22^

A limitation of TrueCa is that its performance decreases at low iodine concentrations, when low density calcium and iodine voxels overlap in the spectral space of Figure 3. Although one might expect a more angled decision boundary to better separate these materials, our optimization yielded a sharper boundary. This reflects partial volume effects at the lumen–vessel wall interface, where a more restrictive threshold was necessary to avoid false positive calcium classification and maintain Agatston scores of zero in uncalcified regions. As a result, TrueCa relies on sufficient iodine enhancement (≥390 HU in this study) for optimal performance, which may restrict applicability in protocols with reduced contrast doses. Future work may explore more flexible nonlinear decision boundaries to improve separation at low iodine levels and potentially reduce the minimum contrast requirement.

This study has three other limitations. First, only a single phantom was used, containing varying densities of hydroxyapatite and iodine concentrations. As such, the TrueCa algorithm will require further optimization to account for the compositional variability of calcified and partially calcified plaques. Secondly, only relatively large, circular calcifications were evaluated, leaving TrueCa's performance for small or eccentric lesions unassessed in this phantom. Lastly, our model performance requires external and in vivo validation on larger sample sizes, as well as further protocol optimization. Specifically, using virtual monochromatic images in TNC CCT and CAC CT reduces beam‐hardening and blooming artifacts, leading to Agatston scores that more closely approximate those from TrueCa CCTA.

## 5 CONCLUSION

From this phantom study we can conclude that the proposed TrueCa approach demonstrates superior detection and quantification of CAC, in contrast to routine VNC reconstructions, which consistently underestimates Agatston scores. Thin‐slice reconstructions further enhanced calcium detectability on CCTA acquisitions, particularly for medium‐ to very low‐density lesions. These results underscore the potential of TrueCa CCTA as a reliable alternative for non‐contrast CAC CT to reduce radiation exposure and improve workflow.

## CONFLICT OF INTEREST STATEMENT

N.R.W. and E.L. are full‐time employees of Philips Healthcare. All other authors declare to have no relevant conflicts of interest.

## ETHICS STATEMENT

This study did not involve human participants, patient data, or animal subjects.

## Data Availability

Research data may be shared upon reasonable request to the authors.
